# *Sedum ichangensis*, a new species of Crassulaceae from Hubei, China

**DOI:** 10.3897/phytokeys.132.35428

**Published:** 2019-10-02

**Authors:** Yu-Bing Wang, Xing-Jun Xiong

**Affiliations:** 1 Key Laboratory of Three Gorges Regional Plant Genetics & Germplasm Enhancement (CTGU)/Biotechnology Research Center, China Three Gorges University, Yichang, 443002, China China Three Gorges University Yichang China; 2 Changyang Hospital of Traditional Chinese Medicine, Changyang, 443500, China Changyang Hospital of Traditional Chinese Medicine Changyang China

**Keywords:** Crassulaceae, Flora of China, Flora of Hubei, new species, *Sedum* sect. *Filipes*

## Abstract

*Sedum ichangensis***sp. nov.**, from Yichang, Hubei province, central China, is described and illustrated. The new species is similar to *S. elatinoides* and *S. rosthornianum* in its leaf and carpel morphology and differs in its creeping stems and solitary flowers. The conservation status of *S. ichangensis* was assessed as Endangered according to the IUCN Red List criteria.

## Introduction

*Sedum*Linnaeus (1753: 430), the largest genus in the family Crassulaceae with about 430 species, is particularly diverse in East Asia, the Mediterranean and North America (Linnaeus 1753; ’t Hart and Bleij 2003; Thiede and Eggli 2007). *Sedum* can easily be distinguished by its usually alternate leaves, sessile carpels with slightly connate at the base, separate, mostly yellow or white petals and stamens with two whorls (Thiede and Eggli 2007); however, molecular studies have revealed that *Sedum* is a highly polyphyletic group (Nikulin et al. 2016) which may be due to the high morphological plasticity and variability within the genus (Carrillo-Reyes et al. 2009). In China, 121 species were recorded in the "Flora of China" (Fu and Ohba 2001). Since 2001, six new species have been described, namely *S. hoi* X. F. Jin & B. Y. Ding (2005: 381), *S. spiralifolium* D.Q. Wang, D.M. Xie & L Q. Huang (2014: 117), *S. plumbizincicola* X.H. Guo & S.B. Zhou ex L.H. Wu (2013: 492), *S. fanjingshanensis* C. D. Yang & X. Y. Wang (2012: 389), *S. kuntsunianum* X. F. Jin, S. H. Jin & B. Y. Ding (2013: 34) and *S. peltatum* M. L. Chen & X. H. Cao (2017: 847).

According to the recent taxonomic treatment of Fu and Ohba (2001), the species of *Sedum* in China are divided into three sections, viz. Sedum sect. Sedum, sect. Oreades (Fröderström) K.T. Fu and sect. Filipes (Fröderström) K.T. Fu. The section Sedum is distinct from both sections *Oreades* and *Filipes* in its adaxially gibbous carpels and follicles (vs. carpels and follicles not gibbous); while the sect. Oreades differs from the sect. Filipes in its spurred (vs. spurless) leaf base and petals that are mainly yellow (vs. mainly white or reddish-purple) (Fu and Ohba 2001).

An unknown Sedum species, belonging to the sect. Filipes, was discovered in Hubei Province, Central China. The species is described as new to science in this study.

## Material and methods

Three scattered populations of an unknown *Sedum* species were discovered in Yichang city of Hubei Province, Central China in 2014. These populations were continuously observed over 2 years. Fresh specimens collected from these populations were morphologically studied and illustrated. The distribution map was constructed with Arcgis 10.2, using data provided on the specimen labels.

Specimens of the morphologically similar species *Sedum elatinoides*Franchet (1883: 11) and *S. rosthornianum* Diels (1900: 361) were collected from Hubei province for comparison. Specimens of Sedum sect. Filipes deposited at PE, HIB, WH and CCAU were largely checked, based on the relevant literature (Fu and Ohba 2001, Fu 2001). Furthermore, digital images of type specimens archived at the JSTOR Global Plants website (http://plants.jstor.org) were examined.

## Taxonomy

### Sedum ichangensis

Taxon classificationPlantaeSaxifragalesCrassulaceae

Y. B. Wang sp. nov.

8E152EB5-6268-5CE2-B924-39BBE84F5367

urn:lsid:ipni.org:names:60479381-2

Figures 1 , 2

#### Diagnosis.

**Figure 1.**
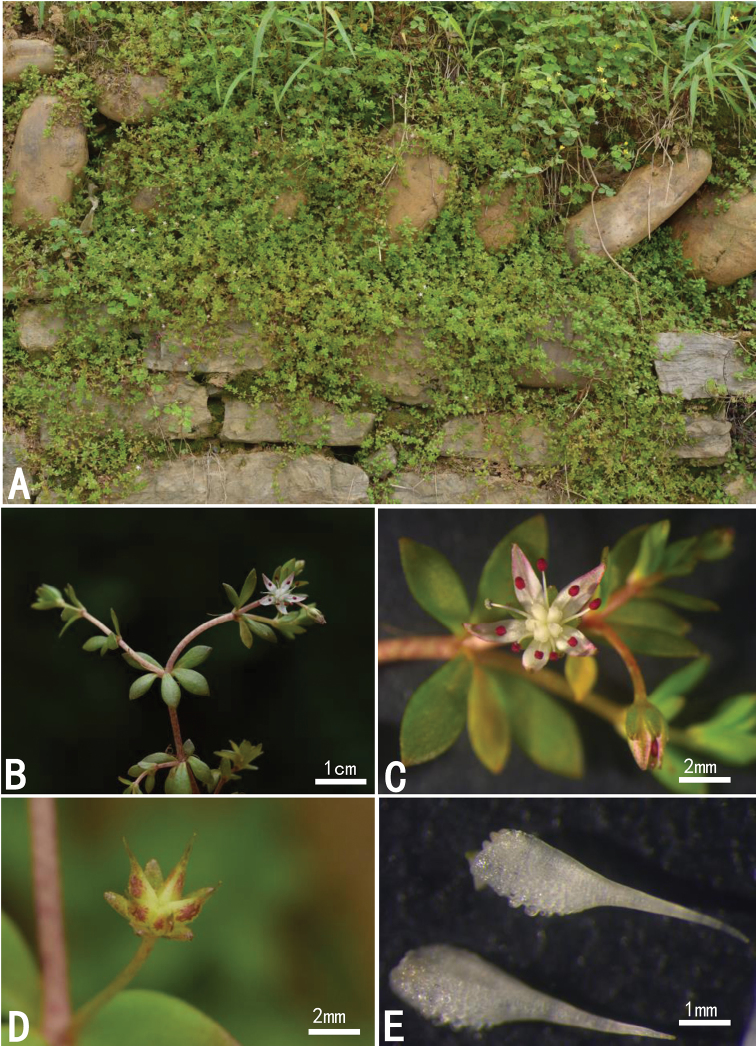
*Sedum ichangensis* Y. B. Wang from type locality **A** habitat **B** flowering stems **C** flower **D** unripe follicles **E** carpels with style.

**Figure 2.**
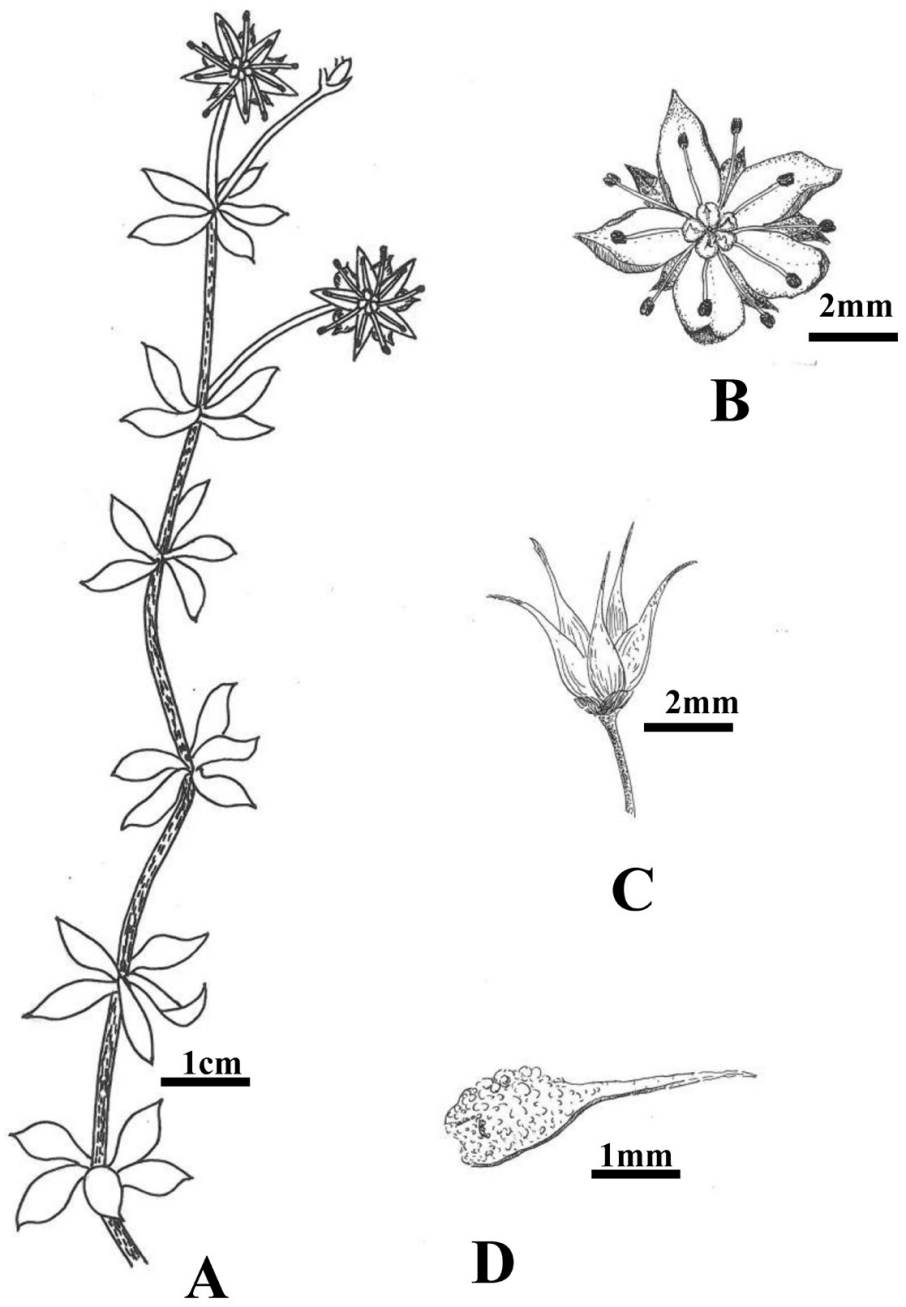
*Sedum ichangensis* Y. B. Wang, sp. nov. **A** habit **B** flower **C** carpels **D** single carpel.

*Sedum ichangensis* has papillate carpels and appears to be morphologically similar to *S. elatinoides* and *S. rosthornianum*. It can be distinguished from *S. elatinoides* by its perennial habit (vs. annual) and solitary flower (vs. flowers in cymes) and from *S. rosthornianum* in its entire leaf margins (vs. leaf margins dentate), its branched stems (vs. stems simple) and its solitary flowers (vs. flowers in paniculiform cymes). (Table 1, Fig. 1, 2).

**Table 1.** Morphological comparison between *Sedum ichangensis* and related species.

**Item**	***Sedum ichangensis***	***Sedum elatinoides***	***Sedum rosthornianum***
**Habit**	perennial	annual	perennial
**Stem**	prostrate	erect	erect
**Phyllotaxis**	4–6-verticillate	3–6-verticillate	opposite or 3- or 4-verticillate
**Leaf blade**	narrowly ellipsoid, entire	narrowly oblanceolate, entire	rhombic-oblong, dentate
**Inflorescence**	solitary flower	paniculiform or corymbiform cyme	paniculiform cyme
**Petal**	white, pinkish toward the apex	white	white

#### Type.

**CHINA**: Hubei Province, Yichang city, Changyang County, Longzhouping Town, on rocks, alt. 130 m. 30°28'N, 111°11'E, 19 Jul 2017, ycmy032 (holotype, CTGU!; isotypes HIB!, and PE!).

#### Description.

Perennial herbs. Roots fibrous. Stems procumbent, divaricately branched, 1–2 mm in diameter, up to 35 cm long, with scattered reddish dots. Leaves 4–6-verticillate, entire, sessile, narrowly ellipsoid, 5–12 × 1.5–2.5 mm, base attenuate, apex acute. Flowers 5-merous, solitary in the axils of upper leaves, 5–8 mm in diameter. Pedicel 1.5–2.5 cm long. Sepals 5, lanceolate, 1.5–2 mm long, apex acute. Petals 5, white, pinkish towards the apex, lanceolate, 4–5 × 1–2 mm, apex acute. Stamens 10, in 2 whorls, slightly shorter than the petals, antesepalous ones ca. 4 mm long, antepetalous ones ca. 3 mm long, inserted ca. 1 mm above the petal base, filaments white, 1.6–2.4 mm long, anthers ca. 0.4 mm long, reddish. Nectar scales spatulate, ca. 0.4 mm long. Carpels 5, white, suberect, adaxially minutely papillate, broadly ovoid, ca. 2 mm long, base united for ca. 0.2 mm, styles ca. 1.5 mm long. Follicles divergent, 0.8–1.1 mm long, with scattered reddish dots, seeds numerous, brown, ca. 0.5–1 mm long, papillate.

#### Phenology.

Flowering from early May to July, fruiting from August to October.

#### Distribution and habitat.

**Figure 3.**
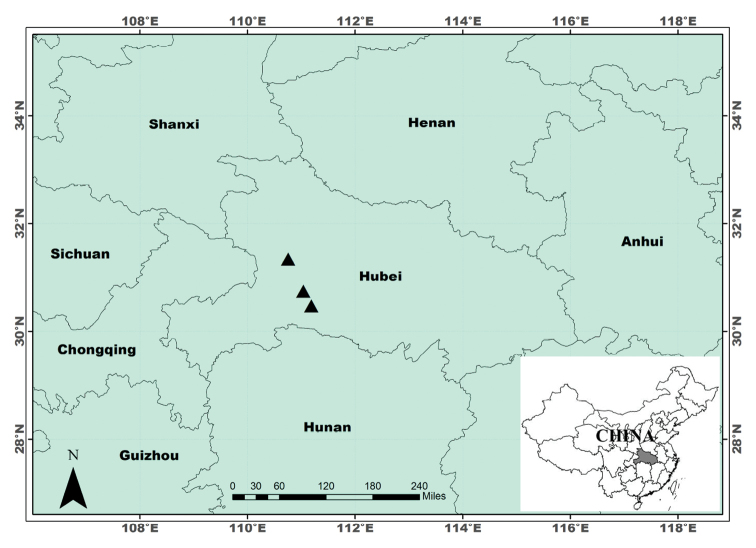
Distribution of *Sedum ichangensis* in Hubei province, central China. The three known localities are indicated with triangles.

*Sedum ichangensis* is known from Longzhouping town of Changyang County, Gufu town of Xingshan County and Muyang River of Yiling County in Yichang City of western Hubei Province, central China (Fig. 3). It grows on rocks of roadsides, especially in fissures filled with soil, at an elevation of ca. 100–280 m.

#### Chinese name.

Yi-chang-jing-tian (宜昌景天).

#### Etymology.

The specific epithet of this new species is dedicated to the Yichang city.

#### Taxonomic notes.

*Sedum ichangensis* belongs to Sedum sect. Filipes on account of its carpels adaxially not gibbous, its spurless leaf base and its white flowers (Fu and Ohba 2001). *Sedum ichangensis* is a species easily identifiable by its floral, stem and leaf features. The new species resembles *S. elatinoides* in the leaf characters, as well as the structure of the flowers. However, *S. ichangensis* differs from *S. elatinoides* in its perennial habit with branched stems and its solitary flowers. *Sedum ichangensis* differs from *S. rosthornianum* in its much branched, decumbent stems, entire leaf margins and its solitary flowers. Here, we provide photographs (Fig. 1), line drawings (Fig. 2) and a detailed morphological comparison (Table 1), as well as a key to the species of Sedum sect. Filipes in China to facilitate its identification.

#### Additional specimens examined (paratypes).

CHINA. Hubei Province: Xingshan County, Gufu town, 200 m alt., 31°20'N, 110°45'E, 15 May 2017, YB Wang ycmy022 (CTGU), same loc. XJ Xiong XXJ024 (CTGU); Yiling County, Muyang River, 280 m alt., 30°44'N, 111°02'E, 3 August 2017, YB Wang ycmy139 (CTGU).

#### Conservation status.

Based on field investigations, *S. ichangensis* occurs only in three scattered areas. The total area of occupancy is less than 500 km^2^; each population possesses no more than 300 mature individuals. It prefers habitats on rocks along roads. Human activities are impairing its populations severely. The type population, which grew close to a road, was seriously impacted in its survival due to herbicide spraying in 2018. Based on currently available information, the conservation status of this species is categorised as Endangered [EN] following the IUCN Categories and Criteria (IUCN 2017).

##### Key to the species of Sedum sect. Filipes in China (adapted from Fu and Ohba 2001):

**Table:** 

1	Plants perennial, fasciculate; stamens in 1 series	***S. correptum***
–	Plants annual or biennial, rarely perennial, solitary or tufted; stamens in 2 series	**2**
2	Plants glandular hairy	**3**
–	Plants glabrous.	**4**
3	Plants annual; stems soft; leaves 2–4 × 1.4–2.5 cm	***S. drymarioides***
–	Plants biennial; stems ± woody at base; leaves 0.7–1.5 × 0.7–0.9 cm	***S. stellariifolium***
4	Carpels minutely papillate	**5**
–	Carpels smooth	**7**
5	Stems simple, erect; leaf margin dentate	***S. rosthornianum***
–	Stems many branched, decumbent; leaf margin entire	**6**
6	Plants annual; stems erect, flowers in cymes	***S. elatinoides***
–	Plants perennial, stems creeping, flowers solitary	***S. ichangensis***
7	Carpels 3	***S. bonnieri***
–	Carpels 5	**8**
8	Petals reddish-purple; flowering stems branched, ca. 20 cm	***S. filipes***
–	Petals white; flowering stems simple, ca. 10 cm	***S. majus***

## Supplementary Material

XML Treatment for Sedum ichangensis
